# Supplementary material for: Modeling glioblastoma heterogeneity as a dynamic network of cell states
Source: Mol Syst Biol. 2021 Sep 16;17(9):e10105. doi: 10.15252/msb.202010105 (PMC8444284; doi:10.15252/msb.202010105)
Supplement: Supplementary file 5 — Source Data for Figure 3 [file MSB-17-e10105-s001.zip › Figure3A_sourcedata/GSEA_3065/hallmarks_state4.GseaPreranked.1623416296068/HALLMARK_MITOTIC_SPINDLE.html]

Details for gene set HALLMARK\_MITOTIC\_SPINDLE[GSEA]

|  || Dataset | state4 |
| Phenotype | NoPhenotypeAvailable |
| Upregulated in class | na\_neg |
| GeneSet | HALLMARK\_MITOTIC\_SPINDLE |
| Enrichment Score (ES) | -0.61401385 |
| Normalized Enrichment Score (NES) | -2.3041458 |
| Nominal p-value | 0.0 |
| FDR q-value | 0.0 |
| FWER p-Value | 0.0 |
Table: GSEA Results Summary

  

Fig 1: Enrichment plot: HALLMARK\_MITOTIC\_SPINDLE      
 Profile of the Running ES Score & Positions of GeneSet Members on the Rank Ordered List

  

| PROBE | GENE SYMBOL | GENE\_TITLE | RANK IN GENE LIST | RANK METRIC SCORE | RUNNING ES | CORE ENRICHMENT || 1 | MARCKS |  |  | 27 | 0.363 | 0.0136 | No |
| 2 | PCGF5 |  |  | 161 | 0.216 | 0.0026 | No |
| 3 | KIF3B |  |  | 379 | 0.158 | -0.0250 | No |
| 4 | FSCN1 |  |  | 428 | 0.147 | -0.0255 | No |
| 5 | KIF1B |  |  | 437 | 0.145 | -0.0196 | No |
| 6 | ARL8A |  |  | 520 | 0.134 | -0.0264 | No |
| 7 | CDC42EP4 |  |  | 600 | 0.125 | -0.0330 | No |
| 8 | YWHAE |  |  | 724 | 0.111 | -0.0476 | No |
| 9 | WASL |  |  | 770 | 0.106 | -0.0497 | No |
| 10 | RALBP1 |  |  | 834 | 0.101 | -0.0550 | No |
| 11 | CLIP2 |  |  | 836 | 0.101 | -0.0501 | No |
| 12 | RANBP9 |  |  | 947 | 0.092 | -0.0635 | No |
| 13 | APC |  |  | 1029 | 0.086 | -0.0725 | No |
| 14 | ITSN1 |  |  | 1093 | 0.082 | -0.0787 | No |
| 15 | KIF3C |  |  | 1152 | 0.079 | -0.0842 | No |
| 16 | UXT |  |  | 1201 | 0.076 | -0.0883 | No |
| 17 | ARHGAP5 |  |  | 1259 | 0.073 | -0.0940 | No |
| 18 | CCDC88A |  |  | 1279 | 0.072 | -0.0935 | No |
| 19 | KIFAP3 |  |  | 1310 | 0.070 | -0.0949 | No |
| 20 | SPTAN1 |  |  | 1371 | 0.067 | -0.1014 | No |
| 21 | WASF2 |  |  | 1385 | 0.067 | -0.1002 | No |
| 22 | OPHN1 |  |  | 1390 | 0.067 | -0.0975 | No |
| 23 | DYNLL2 |  |  | 1607 | 0.058 | -0.1299 | No |
| 24 | SOS1 |  |  | 1760 | 0.053 | -0.1521 | No |
| 25 | ARHGEF3 |  |  | 1771 | 0.053 | -0.1511 | No |
| 26 | MARK4 |  |  | 1810 | 0.052 | -0.1547 | No |
| 27 | RABGAP1 |  |  | 1814 | 0.052 | -0.1526 | No |
| 28 | PALLD |  |  | 1930 | 0.049 | -0.1690 | No |
| 29 | GSN |  |  | 1935 | 0.049 | -0.1672 | No |
| 30 | ABI1 |  |  | 1956 | 0.048 | -0.1681 | No |
| 31 | PAFAH1B1 |  |  | 1964 | 0.048 | -0.1668 | No |
| 32 | PREX1 |  |  | 2110 | 0.044 | -0.1883 | No |
| 33 | CAPZB |  |  | 2121 | 0.043 | -0.1878 | No |
| 34 | PPP4R2 |  |  | 2151 | 0.043 | -0.1904 | No |
| 35 | BCR |  |  | 2169 | 0.042 | -0.1911 | No |
| 36 | ARHGEF12 |  |  | 2202 | 0.042 | -0.1943 | No |
| 37 | STAU1 |  |  | 2249 | 0.040 | -0.1998 | No |
| 38 | CDC42 |  |  | 2300 | 0.039 | -0.2060 | No |
| 39 | WASF1 |  |  | 2371 | 0.038 | -0.2156 | No |
| 40 | FGD4 |  |  | 2438 | 0.036 | -0.2245 | No |
| 41 | PDLIM5 |  |  | 2517 | 0.034 | -0.2356 | No |
| 42 | MID1 |  |  | 2518 | 0.034 | -0.2339 | No |
| 43 | NEDD9 |  |  | 2524 | 0.034 | -0.2330 | No |
| 44 | ARHGEF7 |  |  | 2587 | 0.033 | -0.2415 | No |
| 45 | VCL |  |  | 2710 | 0.030 | -0.2599 | No |
| 46 | CD2AP |  |  | 2744 | 0.030 | -0.2638 | No |
| 47 | FLNA |  |  | 3061 | 0.024 | -0.3142 | No |
| 48 | STK38L |  |  | 3135 | 0.022 | -0.3250 | No |
| 49 | SPTBN1 |  |  | 3155 | 0.022 | -0.3270 | No |
| 50 | TSC1 |  |  | 3172 | 0.022 | -0.3285 | No |
| 51 | KATNB1 |  |  | 3240 | 0.020 | -0.3385 | No |
| 52 | ABL1 |  |  | 3254 | 0.020 | -0.3396 | No |
| 53 | CSNK1D |  |  | 3263 | 0.019 | -0.3399 | No |
| 54 | ARFGEF1 |  |  | 3273 | 0.019 | -0.3405 | No |
| 55 | NF1 |  |  | 3306 | 0.018 | -0.3448 | No |
| 56 | AKAP13 |  |  | 3405 | 0.015 | -0.3600 | No |
| 57 | LATS1 |  |  | 3454 | 0.014 | -0.3672 | No |
| 58 | HOOK3 |  |  | 3555 | 0.011 | -0.3830 | No |
| 59 | CLIP1 |  |  | 3585 | 0.009 | -0.3873 | No |
| 60 | KLC1 |  |  | 3611 | 0.009 | -0.3909 | No |
| 61 | FARP1 |  |  | 3631 | 0.008 | -0.3936 | No |
| 62 | ABR |  |  | 3660 | 0.007 | -0.3979 | No |
| 63 | MAPRE1 |  |  | 3682 | 0.006 | -0.4010 | No |
| 64 | RASAL2 |  |  | 3859 | -0.002 | -0.4296 | No |
| 65 | ROCK1 |  |  | 3943 | -0.006 | -0.4429 | No |
| 66 | NOTCH2 |  |  | 3944 | -0.006 | -0.4426 | No |
| 67 | TLK1 |  |  | 3945 | -0.006 | -0.4422 | No |
| 68 | TUBGCP2 |  |  | 4167 | -0.020 | -0.4773 | No |
| 69 | CYTH2 |  |  | 4207 | -0.022 | -0.4826 | No |
| 70 | ARHGDIA |  |  | 4309 | -0.030 | -0.4976 | No |
| 71 | CTTN |  |  | 4312 | -0.030 | -0.4965 | No |
| 72 | RFC1 |  |  | 4316 | -0.030 | -0.4954 | No |
| 73 | EZR |  |  | 4342 | -0.032 | -0.4980 | No |
| 74 | SHROOM2 |  |  | 4358 | -0.033 | -0.4988 | No |
| 75 | RASA2 |  |  | 4409 | -0.036 | -0.5052 | No |
| 76 | RICTOR |  |  | 4425 | -0.037 | -0.5057 | No |
| 77 | SORBS2 |  |  | 4585 | -0.050 | -0.5292 | No |
| 78 | TUBGCP3 |  |  | 4643 | -0.055 | -0.5358 | No |
| 79 | ARHGAP10 |  |  | 4830 | -0.070 | -0.5627 | No |
| 80 | MYH10 |  |  | 4880 | -0.074 | -0.5670 | No |
| 81 | ACTN4 |  |  | 5044 | -0.086 | -0.5894 | No |
| 82 | EPB41 |  |  | 5162 | -0.098 | -0.6036 | No |
| 83 | PCM1 |  |  | 5177 | -0.099 | -0.6009 | No |
| 84 | MYO9B |  |  | 5234 | -0.104 | -0.6049 | No |
| 85 | DST |  |  | 5242 | -0.105 | -0.6008 | No |
| 86 | RHOT2 |  |  | 5253 | -0.106 | -0.5972 | No |
| 87 | CEP192 |  |  | 5351 | -0.114 | -0.6074 | No |
| 88 | SASS6 |  |  | 5352 | -0.114 | -0.6017 | No |
| 89 | CNTRL |  |  | 5363 | -0.115 | -0.5977 | No |
| 90 | KIF5B |  |  | 5464 | -0.127 | -0.6077 | Yes |
| 91 | CEP250 |  |  | 5465 | -0.127 | -0.6014 | Yes |
| 92 | SUN2 |  |  | 5493 | -0.131 | -0.5994 | Yes |
| 93 | NEK2 |  |  | 5505 | -0.132 | -0.5946 | Yes |
| 94 | CEP57 |  |  | 5548 | -0.136 | -0.5947 | Yes |
| 95 | CKAP5 |  |  | 5549 | -0.136 | -0.5880 | Yes |
| 96 | PCNT |  |  | 5575 | -0.140 | -0.5851 | Yes |
| 97 | DLG1 |  |  | 5592 | -0.144 | -0.5806 | Yes |
| 98 | CENPJ |  |  | 5625 | -0.147 | -0.5785 | Yes |
| 99 | EPB41L2 |  |  | 5665 | -0.154 | -0.5772 | Yes |
| 100 | TTK |  |  | 5684 | -0.157 | -0.5724 | Yes |
| 101 | KNTC1 |  |  | 5703 | -0.161 | -0.5673 | Yes |
| 102 | DYNC1H1 |  |  | 5710 | -0.162 | -0.5603 | Yes |
| 103 | CDK5RAP2 |  |  | 5798 | -0.182 | -0.5654 | Yes |
| 104 | ARHGAP29 |  |  | 5807 | -0.184 | -0.5576 | Yes |
| 105 | SAC3D1 |  |  | 5810 | -0.186 | -0.5487 | Yes |
| 106 | PRC1 |  |  | 5835 | -0.192 | -0.5431 | Yes |
| 107 | PLK1 |  |  | 5836 | -0.192 | -0.5336 | Yes |
| 108 | TRIO |  |  | 5840 | -0.193 | -0.5245 | Yes |
| 109 | MYH9 |  |  | 5874 | -0.200 | -0.5200 | Yes |
| 110 | BRCA2 |  |  | 5910 | -0.210 | -0.5153 | Yes |
| 111 | RACGAP1 |  |  | 5953 | -0.223 | -0.5111 | Yes |
| 112 | KIF2C |  |  | 5996 | -0.237 | -0.5062 | Yes |
| 113 | SMC1A |  |  | 6017 | -0.245 | -0.4973 | Yes |
| 114 | AURKA |  |  | 6026 | -0.248 | -0.4863 | Yes |
| 115 | SMC3 |  |  | 6041 | -0.265 | -0.4755 | Yes |
| 116 | BUB1 |  |  | 6045 | -0.266 | -0.4627 | Yes |
| 117 | FBXO5 |  |  | 6084 | -0.287 | -0.4547 | Yes |
| 118 | KIF22 |  |  | 6089 | -0.290 | -0.4410 | Yes |
| 119 | PIF1 |  |  | 6106 | -0.301 | -0.4287 | Yes |
| 120 | KIF11 |  |  | 6109 | -0.304 | -0.4139 | Yes |
| 121 | LMNB1 |  |  | 6134 | -0.325 | -0.4017 | Yes |
| 122 | CCNB2 |  |  | 6140 | -0.329 | -0.3862 | Yes |
| 123 | NDC80 |  |  | 6142 | -0.330 | -0.3700 | Yes |
| 124 | DLGAP5 |  |  | 6156 | -0.345 | -0.3550 | Yes |
| 125 | KIF15 |  |  | 6158 | -0.346 | -0.3380 | Yes |
| 126 | KIF23 |  |  | 6162 | -0.351 | -0.3210 | Yes |
| 127 | KIF4A |  |  | 6164 | -0.355 | -0.3036 | Yes |
| 128 | KIF20B |  |  | 6172 | -0.373 | -0.2862 | Yes |
| 129 | ECT2 |  |  | 6178 | -0.382 | -0.2681 | Yes |
| 130 | BIRC5 |  |  | 6214 | -0.484 | -0.2498 | Yes |
| 131 | ANLN |  |  | 6215 | -0.484 | -0.2257 | Yes |
| 132 | SMC4 |  |  | 6219 | -0.490 | -0.2019 | Yes |
| 133 | NUSAP1 |  |  | 6223 | -0.507 | -0.1772 | Yes |
| 134 | CENPE |  |  | 6226 | -0.511 | -0.1522 | Yes |
| 135 | CDK1 |  |  | 6246 | -0.627 | -0.1242 | Yes |
| 136 | TPX2 |  |  | 6253 | -0.667 | -0.0921 | Yes |
| 137 | TOP2A |  |  | 6260 | -0.814 | -0.0527 | Yes |
| 138 | CENPF |  |  | 6263 | -1.068 | 0.0000 | Yes |
Table: GSEA details [plain text format]

  

Fig 2: HALLMARK\_MITOTIC\_SPINDLE: Random ES distribution      
 Gene set null distribution of ES for **HALLMARK\_MITOTIC\_SPINDLE**
